# Hydrothorax as a Complication of Peritoneal Dialysis: A Case Report

**DOI:** 10.1155/crin/4365717

**Published:** 2025-11-30

**Authors:** Luca Piscitani, Paolo Sipari, Vittorio Di Michele, Lorenzo Ottavio Di Pietro, Vittorio Sirolli, Marilena Tunno

**Affiliations:** ^1^Nephrology and Dialysis Unit, Department of Medicine, San Salvatore Hospital, Via Lorenzo Natali n°1, L'Aquila 67100, Italy; ^2^Department of Clinical Medicine, Public Health, Life and Environmental Sciences, University of L'Aquila, Via Vetoio, L'Aquila 67100, Italy; ^3^Nephrology and Dialysis Unit, Department of Medicine, G. d'Annunzio University of Chieti-Pescara, Chieti 66100, Italy

**Keywords:** hydrothorax, peritoneal CT, peritoneal dialysis

## Abstract

Hydrothorax is a possible complication of peritoneal dialysis due to migration of dialysate from the peritoneal cavity to the thoracic cavity through a congenital or acquired diaphragmatic defect that allows its passage. Diagnosis is clinical, supported by peritoneal CT and pleural fluid analysis. Therapy is given in the first instance by discontinuation of the peritoneal method, but sometimes an attempt may be made to reinstate it with appropriate changes in the prescription. We report here our experience about a case of hydrothorax during peritoneal dialysis reporting the most recent protocols for proper management.

## 1. Introduction

Hydrothorax is a medical condition characterized by the accumulation of serous fluid in the peritoneal cavity. It is a possible complication of peritoneal dialysis due to the migration of dialysate from the peritoneal cavity to the thoracic cavity through a congenital or acquired diaphragmatic defect that allows its passage [1]. It generally develops on the right side, due to increased intraperitoneal pressure, and the incidence ranges from 1.6% to 10% in peritoneal dialysis patients [2]. In addition to the point of passage in the diaphragm, another essential factor for the development of this complication is the pressure difference between the abdominal cavity (which is greater due to the presence of the dialysate fluid) and the thoracic cavity, which is by definition negative in order to allow lung expansion [3].

Pleuroperitoneal communications are abnormal connections between the pleural and peritoneal cavities. The risk factors and pathophysiology of pleuroperitoneal communications are congenital defects, such as diaphragmatic hernias or defects in the diaphragm, which can create a communication between the pleural and peritoneal cavities; anatomical abnormalities in the diaphragm, such as eventration or paralysis, which can increase the risk of pleuroperitoneal communications; and conditions such as ascites, pregnancy, or obesity that can increase the intra-abdominal pressure, leading to the development of pleuroperitoneal communications [4–6].

The patients undergoing peritoneal dialysis are at risk of developing pleuroperitoneal communications due to the increased pressure and fluid flow in the peritoneal cavity.

The pathophysiology of pleuroperitoneal communications involves a pressure gradient between the peritoneal and pleural cavities, diaphragmatic defects, and abnormalities in lymphatic drainage, which collectively facilitate fluid flow and accumulation in the pleural space [7].

In the clinical case we are about to describe, we will discuss a patient undergoing peritoneal dialysis who developed hydrothorax, focusing in particular on the diagnostic strategy, the therapies progressively implemented, and the attempt to maintain the method.

## 2. Case Report

**Figure 1:**
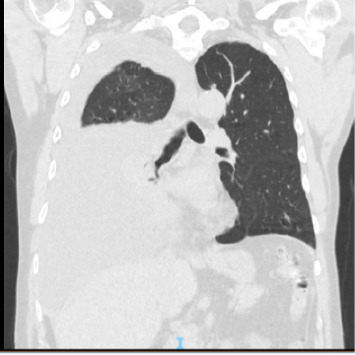
Chest CT scan showing severe unilateral pleural effusion.

**Figure 2:**
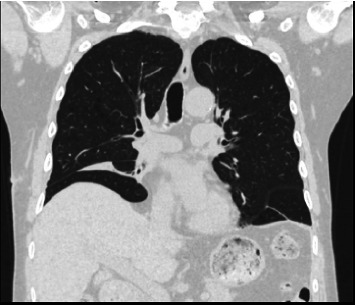
Chest CT scan following evacuation thoracentesis.

**Figure 3:**
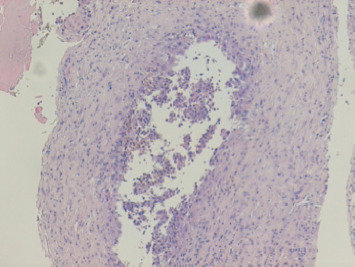
Pleural biopsy sample undergoing histological examination (hematoxylin–eosin).

**Figure 4:**
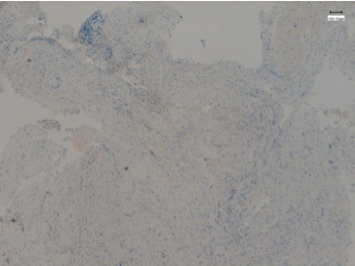
Pleural biopsy sample undergoing histological examination (P40 negative).

**Figure 5:**
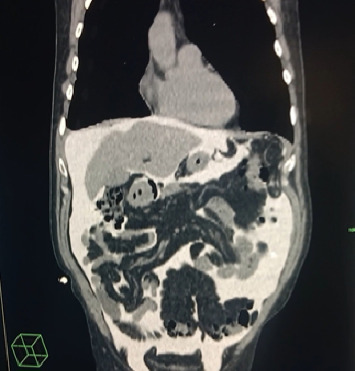
Peritoneal CT scan after restoration of the peritoneal method.

**Figure 6:**
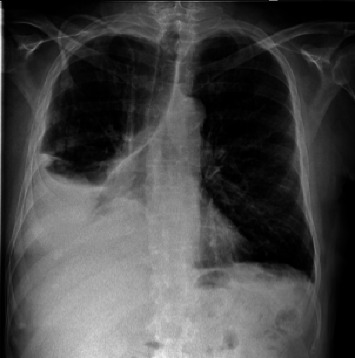
X-ray showing recurrence of hydrothorax.

A 64-year-old man suffering from chronic renal failure secondary to Alport syndrome diagnosed in 2021 was undergoing treatment for systemic arterial hypertension, hypothyroidism, benign prostatic hyperplasia, and hyperuricemia, for which he was on levothyroxine, amlodipine, ramipril, ramsulosin, and allopurinol. In March 2021, a peritoneal catheter was placed to begin peritoneal dialysis treatment. After appropriate training for automated peritoneal dialysis (APD) and blood chemistry tests documenting end-stage chronic renal failure (creatinine 9.7 mg/dL, blood urea nitrogen 158 mg/dL, sodium 140 mEq/L, and potassium 4.8 mEq/L), the patient began APD treatment with glucose 1.36% bags and 75% tydal in May 2021. After a year without problems, the patient experienced an episode of peritoneal catheter obstruction due to fibrin deposition, which was appropriately treated with heparinized solution washings, with good recovery of catheter function. In July 2022, problems were encountered during APD, in particular, a reduction in drainage fluid. Initially, abdominal and chest X-rays were performed, which documented the tip of the catheter in the mesogastrium and mild pleural effusion. Later on, the patient complained of dyspnea and asthenia and was referred to the emergency room. A chest CT scan was performed (Figure 1), which showed severe unilateral pleural effusion. Due to suspected pleuroperitoneal communication, the patient was admitted to the medical ward and underwent evacuative thoracentesis, after which a follow-up chest CT scan was performed (Figure 2). In light of this suspicion, peritoneal dialysis was suspended. Also, after pleural drainage of 3000 cc, the glucose concentration of the fluid, measured using a blood glucose meter, was 144 mg/dL with a serum glucose level of 85 mg/dL, which was consistent with the previously loaded peritoneal fluid. All investigations into the secondary nature of the pleural effusion were negative, including histological analysis of pleural biopsies (Figures 3 and 4). Considering the patient's preserved diuresis and good control of the uremic syndrome resulting from blood chemistry tests, conservative medical therapy was administered in the first instance, with simultaneous serial monitoring of renal function. In August 2022, a temporary central venous catheter was placed in the jugular vein for conversion to hemodialysis, which the patient underwent three times a week for about a month. Given the patient's strong motivation to return to peritoneal dialysis treatment, in September 2022, after a negative chest X-ray, continuous ambulatory peritoneal dialysis (CAPD) was resumed with reduced abdominal loads, with a maximum of 1200 cc for three exchanges per day and an empty abdomen at night. In February 2024, in order to evaluate the restoration of an automated dialysis method, peritoneal CT (Figure 5) was performed according to a specific protocol, as reported in Table 1, in the supine and prone positions and during puncture, which documented regular distribution of the contrast medium in the intraperitoneal space, with no evidence of frank spillage into the thoracic cavity attributable to macroscopic fistulous tracts. A diffuse slight increase in the tomodensitometric values of the pleural effusion, estimated to be approximately between 15 and 30 HU (Table 2), was also documented. Therefore, given the suspicion of a nonmacroscopically evident communication, CAPD was continued. In February 2025, there was a recurrence of hydrothorax, for which the patient was admitted to the respiratory intensive care unit of our hospital. There, the recurrence of hydrothorax was confirmed by chest X-ray (Figure 6), so it was decided to suspend peritoneal dialysis and start hemodialysis treatment, which is still ongoing.

## 3. Discussion

The clinical case presented deals with a case of hydrothorax as a noninfectious complication of peritoneal dialysis, known to be a major cause of dialysis failure [8, 9]. Clinically, the main symptom reported by patients is dyspnea, while other symptoms may include pleural chest pain, reduced dialysate drainage, and reduced dialysis effectiveness [10]. The diagnosis is clinical, and the patient presents with reduced breath sounds at the bases and dullness on percussion; useful complementary tests include CT with peritoneography and pleural fluid analysis [11, 12]. The latter can detect reduced LDH and leukocyte levels and high glucose concentrations compared to serum glucose, with differences exceeding 50 mg/dL [12]. This increase in glucose concentrations is referred to as “sweet hydrothorax.” Treatment is initially conservative, with discontinuation of peritoneal dialysis and temporary transition to hemodialysis. The closure of the diaphragmatic passage point can occur spontaneously or be achieved through pleurodesis, which involves the instillation of sclerosing agents such as talc. In more severe and extensive cases, video-assisted thoracic surgery (VATS) may be used with intraoperative instillation of dialysate to assess the success of the surgery [13].

This article aims to explore this complication, which is still little discussed in the literature, despite its decisive impact on the patient's therapeutic pathway. The patient in our clinical case developed hydrothorax approximately 14 months after starting peritoneal treatment. Studies in literature indicate that most episodes occur within the first year [8], with an average interval of approximately 20 weeks [9], but onset can also occur years later (up to 8 years reported) [8], making the clinical case consistent with the temporal variability described.

These data highlight the importance of prolonged clinical monitoring, even in the absence of suggestive symptoms in the early stages. The symptoms presented by the patient, dyspnea and asthenia, are consistent with the most common clinical manifestations of hydrothorax in peritoneal dialysis. A specific distinguishing feature in patients undergoing this procedure, highlighted both in the clinical case and in literature, is the reduction in drainage fluid [8].

The correct interpretation of these clinical signs allowed for the timely classification of the complication, avoiding a diagnostic delay that could have aggravated the patient's condition. A crucial role in the diagnosis of our case of hydrothorax was played by chest CT radiodiagnostic techniques, which documented severe pleural effusion on the right side, in accordance with the literature [9]. The diagnosis of hydrothorax was also supported by thoracentesis, another key method for analyzing pleural fluid [2].

The decision to perform a thoracentesis, supplemented by targeted biochemical tests, not only confirmed the diagnosis but also provided insight into the underlying pathophysiological mechanism. The high glucose concentration (144 mg/dL) found in the patient's pleural fluid is characteristic of the condition known as “sweet HT” or “sweet hydrothorax,” associated with peritoneal dialysate [2, 8, 9, 14]. A higher concentration of glucose in the pleural fluid (> 100 mg/dL) than in the serum supports the diagnosis of hydrothorax [9]. In the past, methylene blue was instilled into the dialysate, and the pleural fluid was then analyzed, showing a blue coloration. This method is no longer recommended because it is a possible source of chemical peritonitis [15].

The absence of macroscopically visible findings on subsequent peritoneal CT suggests that pleuroperitoneal communications may be minimal or involve lymphatic pathways that are not easily identifiable with standard techniques [9]. The diagnostic complexity of these cases underscores the importance of an integrated clinical approach that combines observation, imaging, and laboratory analysis. Suspending peritoneal dialysis and temporarily switching to hemodialysis is a standard conservative strategy recommended to allow for the possible resolution of the underlying condition that led to the development of hydrothorax [2]. The subsequent attempt to resume PD with reduced volumes is also a conservative approach aimed at reducing intra-abdominal pressure and minimizing the risk of recurrence [8]. In treating our patient, we chose to use CAPD with smaller exchange volumes, although the literature recommends APD over CAPD because the supine position and smaller exchange volumes reduce intra-abdominal pressure [16]. The recurrence of hydrothorax in the patient highlights how conservative measures are not always sufficient, and more definitive interventions are often necessary to allow long-term PD to continue [9].

Other options for resolving recurrent hydrothorax include pleurodesis, often assisted by VATS, or surgical repair of the diaphragmatic defect, with VATS considered the procedure of choice due to its lower invasiveness and high success rate in allowing a return to peritoneal dialysis [2, 9].

## 4. Conclusion

This study aimed to highlight the fundamental importance of flexibility in the management of dialysis patients, adapting therapeutic strategies to individual clinical variability. The case discussed represents a concrete example of how the management of noninfectious complications of PD requires continuous clinical evolution, based on up-to-date evidence and careful risk–benefit analysis.

This case report fits within the context of other publications on the subject in order to increase awareness of hydrothorax, which remains an important cause of dropout from PD. In this study, we aimed to summarize the available protocols for pleural fluid analysis, dedicated imaging, and possible therapeutic options.

## Figures and Tables

**Table 1:** Protocol for performing peritoneal CT scan.

PERITONEAL CT protocol
(1) Drain the peritoneal cavity;
(2) Perform a baseline chest and abdomen CT scan;
(3) Sterilely add 100 ml of nonionic contrast medium (containing 300 mg of iodine per ml) such as iopromide (ULTRAVIST) or iodixanol (VISIPAQUE) to a 2000-ml bag of dialysate;
(4) Perform peritoneal cavity loading with the bag mixed with the contrast medium;
(5) Ask the patient to walk around to ensure even distribution of the contrast medium solution in the peritoneal cavity;
(6) Perform a thoracoabdominal CT scan after 4 hours of rest;
(7) Drain the peritoneal cavity.

**Table 2:** Diagnostic investigations with related results.

Chemical–physical analysis of drainage fluid	pH 7.29 Hb 0.3 g/dL
Cytological analysis of drainage fluid	No evidence of atypical epithelial elements
Microbiological analysis of drainage fluid	Negative
Microbiological analysis of pleural fluid	Negative
VAT	Chronic nonspecific pleurisy
Pleural biopsies	Negative for neoplasms

## Data Availability

The data that support the findings of this study are available from the corresponding author upon reasonable request.
